# Hypoxia-Induced Differences in the Expression of Pyruvate Dehydrogenase Kinase 1-Related Factors in the Renal Tissues and Renal Interstitial Fibroblast-like Cells of Yak (Bos Grunniens)

**DOI:** 10.3390/ani14213110

**Published:** 2024-10-29

**Authors:** Manlin Zhou, Jun Wang, Ruirui Cao, Fan Zhang, Xuehui Luo, Yiyuan Liao, Weiji Chen, Haie Ding, Xiao Tan, Zilin Qiao, Kun Yang

**Affiliations:** 1 Engineering Research Center of Key Technology and Industrialization of Cell-Based Vaccine, Ministry of Education, Northwest Minzu University, Lanzhou 730030, China; 15888533443@163.com (M.Z.); 15174462439@163.com (F.Z.); 17720068021@163.com (Z.Q.); 2 Gansu Tech Innovation Center of Animal Cell, Biomedical Research Center, Northwest Minzu University, Lanzhou 730030, China; 3 Key Laboratory of Biotechnology and Bioengineering of State Ethnic Affairs Commission, Biomedical Research Center, Northwest Minzu University, Lanzhou 730030, China; 4 College of Life Science and Engineering, Northwest Minzu University, Lanzhou 730030, China; wangjun200005@126.com (J.W.); crr18765529753@outlook.com (R.C.); 15388773298@163.com (X.L.); 13625992622@163.com (Y.L.); zml1527803929@163.com (H.D.); zml15888533443@163.com (X.T.)

**Keywords:** yak, kidney, renal fibrosis, PDK1, TGF-β1/Smad signaling pathway

## Abstract

Hypoxia is one of the factors severely affect renal function, and, in severe cases, it can lead to renal fibrosis. Although much progress has been made in identifying the molecular mediators of fibrosis, the mechanisms that govern renal fibrosis remain unclear, and there have been no effective therapeutic anti-fibrotic strategies to date. Mammals exposed to low oxygen in the plateau environment for a long time are prone to high-altitude disease, while yaks have been living in the plateau for generations do not develop kidney fibrosis caused by low oxygen. It has been suggested that metabolic reprogramming occurs in renal fibrosis and that pyruvate dehydrogenase kinase 1 (PDK1) plays a crucial role in metabolic reprogramming as an important node between glycolysis and the tricarboxylic acid cycle. The aim of this study was to investigate the effects of hypoxia on the renal tissues and renal interstitial fibroblasts of yaks. We found that, at the tissue level, HIF-1α, PDK1, TGF-β1, Smad2, Smad3, and α-SMA were mainly distributed and expressed in tubular epithelial cells but were barely present in the renal mesenchymal fibroblasts of healthy cattle and yak kidneys. Anoptical density analysis showed that in healthy cattle kidneys, TGF-β1, Smad2, and Smad3 expression was significantly higher than in yak kidneys (*p* < 0.05), and HIF-1α and PDK1 expression was significantly lower than in yak kidneys (*p* < 0.05). The results at the protein and gene levels showed the same trend. At the cellular level, prolonged hypoxia significantly elevated PDK1 expression in the renal mesangial fibroblasts of cattle and yak kidneys compared with normoxia (*p* < 0.05) and was proportional to the degree of cellular fibrosis. However, PDK1 expression remained stable in yaks compared with renal interstitial fibroblast-like cells in cattle during the same hypoxic time period. At the same time, prolonged hypoxia also promoted changes in cellular phenotype, promoting the proliferation, activation, glucose consumption, lactate production, and anti-apoptosis in the both of cattle and yaks renal interstitial fibroblasts The differences in kidney structure and expression of PDK1 and HIF-1α in kidney tissue and renal interstitial fibroblasts induced by different oxygen concentrations suggest that there may be a regulatory relationship between yak kidney adaptation and hypoxic environment at high altitude. This provides strong support for the elucidation of the regulatory relationship between PDK1 and HIF-1α, as well as a new direction for the treatment or delay of hypoxic renal fibrosis; additionally, these findings provide a basis for further analysis of the molecular mechanism of hypoxia adaptation-related factors and the adaptation of yaks to plateau hypoxia.

## Simple Summary

Long-term exposure of mammals to the low-oxygen environment of the plateau predisposes them to plateau sicknesses, such as plateau erythrocytosis, pulmonary hypertension, and right ventricular hypertrophy, which are the most common chronic plateau sicknesses in the resident plateau population. Hypoxia is one of the factors that seriously affects renal function and leads to renal fibrosis in severe cases. In contrast, renal fibrosis does not occur in plateau-dwelling yaks, but the underlying molecular mechanisms are unknown. In this manuscript, we compared the histologic differences between the kidneys of cattle and yaks and the changes in PDK1, HIF-1α, and related factors in the renal interstitial fibroblasts of cattle and yaks at different oxygen concentrations (10% and 20%). The results showed significant differences in the histologic structures of yaks and cattle, and PDK1, HIF-1α, and related factors were significantly different expressed in the kidney tissues and renal interstitial fibroblasts of yaks and cattle. This may be related to the adaptation of yak kidney tissues to the low-oxygen environment of plateau, which provides basic information for further analysis of the molecular mechanism of hypoxia adaptation-related factors and the adaptation of yaks to plateau hypoxia.

## 1. Background

The Tibetan Plateau has the highest average altitude and is the largest plateau in the world, characterized by a low temperature, low oxygen, and intense ultraviolet radiation [1]. Oxygen is indispensable for animal growth and development, and low oxygen is one of the greatest challenges to the survival of plateau animals. A lower partial pressure of oxygen leads to an insufficient supply of oxygen the tissues of the body, and this stimulus causes acute or chronic responses in animals affect physiological functions [2]. Mammals exposed to the low-oxygen environment of the plateau for a long period of time are prone to develop plateau diseases, represented by hypoxia-induced renal disease [3], pulmonary hypertension [4], and right ventricular hypertrophy [5]. Moreover, animals inhabiting the plateau have formed unique adaptive structures and physiological mechanisms after a long period of evolution. As a typical representative of the livestock endemic to the Tibetan Plateau, yaks can adapt to its low-temperature and low-oxygen environment due to long-term natural and artificial selection [1]. It also provides a large amount of high-protein food and labor for the local people. Some studies have found that yaks have anatomical and physiological characteristics that can be stably inherited in response to hypoxia adaptation, such as a larger heart and lungs, thicker body coverings, and non-functional sweat glands [6,7]. These characteristics allow them to survive in this extremely harsh environment, which is characterized by cold temperatures, high UV levels, and oxygen deprivation at high altitudes [8,9,10]. Yaks and cattle are distinct species that diverged 5 million years ago; however, they have both strong genomic similarities and extreme differences in their living environments. Thus, examining these two species can shed light on the mechanisms of high-altitude adaptation [11]. In other species, it has been found that the kidney responds to hypoxia through various mechanisms, including the regulation of the acid–base balance, the promotion of angiogenesis, and the management of water and salt metabolism [12,13]. Yak kidneys are also well adapted to the low-temperature and low-oxygen environment of the Tibetan Plateau due to long-term natural selection. They are characterized by a relative thickening of the smooth muscle of the renal arterial wall and specific remodeling of the arterial wall. However, the specific mechanism of how yak kidneys respond to a hypoxic environment still needs to be further explored. Therefore, studying the hypoxic adaptability of the yak kidney is helpful to reveal how it maintains normal functioning under high-altitude conditions, thus ensuring the metabolic adaptability and health of the whole body.

In 1998, Fine et al. made the first conjecture that hypoxia is one of the factors involved in the progression of chronic kidney disease [14]. Recent studies have also shown that hypoxia is a key factor in promoting renal fibrosis. The absence of renal microvasculature, reduced oxygen dispersion, and abnormal cellular metabolism are the main causes of the hypoxic state [15]. Renal fibrosis is a common pathological feature of almost all advanced renal diseases with different etiologies [16]. The main manifestation of renal fibrosis is the excessive synthesis and deposition of interstitial extracellular matrix components, which is associated with the release of inflammatory cytokines, injury to renal tubule epithelial cells (RTECs), the activation and deposition of renal interstitial fibroblast-like cells (RIFs), the development of a new cell culture, the activation and proliferation of fibrosis-related signaling pathways, and the sparing of peritubular microvessels [17,18]. However, previous studies suggest that fibroblasts play a key role in fibrosis [16,19,20]. Activated fibroblasts have been described as myofibroblasts, which are mainly responsible for the production of extracellular matrix (ECM) protein-modifying factors. The exposure of fibroblasts to hypoxic conditions promotes a proliferative fibrotic phenotype and enhances myofibroblast differentiation [15]. Although hypoxia can induce changes in the fibrotic phenotype of fibroblasts, the exact mechanisms underlying this process remain unexplored. Therefore, here, we focus on the effect of renal interstitial fibroblasts on renal fibrosis. Although the exact mechanism of the renal fibrosis process is still unknown, it has been suggested that the dysregulation of transforming growth factor-β1 (TGF-β1) signaling is involved in the hypoxia-induced renal fibrosis process [21,22,23,24].

Recently, a metabolic shift from mitochondrial oxidative phosphorylation to aerobic glycolysis (the Warburg effect) in the kidney has been shown to occur in patients with chronic kidney disease (CKD) and in animal models [25,26,27,28]. Furthermore, the Warburg effect has been shown to promote fibrosis in several other organs, such as the heart and lungs [20,29,30,31]. New evidence suggests that hypoxia-inducible factor-1 α and the hypoxic response play an important role in various types of acute and chronic kidney disease [16]. Hypoxia-inducible factor-1 α (HIF-1α) is an important transcription factor that senses the oxygen status, induces adaptive changes in cellular metabolism, and plays an important role in renal fibrosis and glucose metabolism [17]. Further studies have shown that HIF-1 reprograms metabolism by transcriptionally up-regulating pyruvate dehydrogenase kinase to block the conversion of pyruvate to acetyl-coenzyme A and by regulating the expression of proteins that make up the mitochondrial respiratory chain. Kim and Papandreou [32,33] demonstrated that pyruvate dehydrogenase kinase (PDK)-1 is a direct target gene of HIF-1 and that PDK-1 induction is a key metabolic switch for cellular hypoxia adaptation by increasing glycolysis and inhibiting mitochondrial respiration. This metabolic switch allows hypoxic cells to not only optimize the rate of oxygen consumption but also inhibit the toxic burst of mitochondrial reactive oxygen species [20]. In addition, PDK1 plays an important role in hypoxia-induced fibrosis, and it is involved in a variety of biological functions, such as cell differentiation, migration, apoptosis, and revascularization, by affecting TGF-β/Smad signaling; thus, PDK1 has become the subject of studies examining the occurrence of hypoxia-associated diseases, the mechanism of their development, and their biological treatment [34].

Currently, the animal models of kidney research are mainly focused on mice and rats, and there are still few reports on factors such as PDK1 in the adaptation of the yak kidney to plateau hypoxia. Therefore, in this experiment, histological analysis, RT-qPCR, WB, and other methods were used to detect the histology and the expression distribution of PDK1-related factors in the kidneys of cattle and yaks. And, in conjunction with the biological function of PDK1, it was hypothesized that there is a regulatory relationship between PDK1 and the adaptation of the yak kidney adaptation to the plateau hypoxic environment. The results of this study provide important support for the elucidation of the regulatory relationship between PDK1 and HIF-1α, a new direction for the treatment or delay of hypoxic renal fibrosis, and a basis for further analyzing the molecular mechanism of factors related to hypoxic acclimatization in yaks on the plateau.

## 2. Materials and Methods

### 2.1. Animal Ethics

All animal experiments were approved by the Animal Ethics and Use Committee of the College of Life Sciences and Engineering, Northwest University for Nationalities, under license number DKY-B20151608. All procedures in this study were performed in accordance with the Guidelines for the Care and Use of Laboratory Animals established by the Ministry of Science and Technology of China.

### 2.2. Sample Collection

Kidneys were collected from 3-year-old adult yaks (*n* = 3) in a slaughterhouse in Cooperative City, Gansu province, China (~3500 m above sea level), and from 3-year-old adult cattle (Holstein cow) (*n* = 3) in a slaughterhouse in Tianshan Town, Chifeng City, Inner Mongolia Autonomous Region (~1000 m above sea level). Tissue sample processing: Kidney tissues were cut into 2 cm^3^ pieces and fixed in 4% paraformaldehyde. Molecular sample processing: kidneys were cut into 1 cm^3^ pieces, wrapped in tin foil, transported on dry ice, and placed in a refrigerator at −80 °C for long-term storage. Cellular sample processing: All kidneys were packed in saline at a low temperature and brought back quickly for cell isolation and purification.

### 2.3. Kidney Tissue Sample Processing, Staining and Testing

#### 2.3.1. Paraffin Embedding and Sections

The fixed adult yak and cattle kidney tissues were cut into smooth-edged blocks of 0.5 cm^3^ in volume, put into a fixation box, and rinsed under running water for 24 h. After dehydration and transparency, paraffin embedding was carried out; then, the embedded wax blocks were trimmed and sliced (with a thickness of 5 μm) and used for histological and immunohistochemical staining after spreading, salvaging and baking.

#### 2.3.2. H&E Staining Test

The yak and cattle kidney tissue sections were dewaxed and hydrated by using xylene and gradient alcohol, stained with hematoxylin for 4 min, rinsed under running water for 15 min and then differentiated using 1% hydrochloric acid in alcohol for 2–3 s. The differentiated sections were rinsed under running water for 20 min, then stained with eosin for 6 min for reverse gradient alcohol dehydration and sealed with xylene transparent and neutral resin.

#### 2.3.3. Masson Staining Assay

The yak and cattle kidney tissue sections were dewaxed and hydrated using xylene and gradient alcohol, nucleated with Wiegert ferromyxin for 5–10 min, differentiated with an acidic ethanol differentiation solution for 5–15 s, and then blue with Masson’s bluing solution for 3–5 min. After bluing, they were mass stained with Lichtenstein’s red compound for 5–10 min, washed in a weak acidic working solution for 1 min improve the color of clear fibers, washed in a phosphomolybdic acid staining solution for 1 min, and washed in a weak acid working solution for 1 min for the differentiation and mordanting of collagen fibers. Then, they were stained with an aniline blue solution for 1–2 min and washed with a weak acid working solution for 1 min for the blue staining of collagen fibers. Finally, 95% ethanol and anhydrous ethanol were used to rapidly dehydrate the film, and the film was transparent to xylene 3 times and then sealed with neutral gum after 2 min/times.

#### 2.3.4. Periodic Acid–Schiff (PAS) Staining

The yak and cattle kidney tissue sections were dewaxed and hydrated using xylene and gradient alcohol, then soaked in a chlorination agent for 6 min, rinsed with running water for 15 min, stained with a Schiff dyeing solution away from light for 15 min, rinsed with running water for 10 min, stained with hematoxylin for 1 min, and differentiated with 1% hydrochloric acid alcohol for 2–3 s. The differentiated slices were rinsed with water for 15 min, dehydrated with reverse gradient alcohol, and sealed with xylene transparent and neutral resin.

#### 2.3.5. Immunohistochemical Staining

Immunohistochemical staining was performed using an SP kit to study the expression levels of HIF-1α, PDK1, TGF-β1, Smad2, Smad3 and α-SMA. The kidney tissue sections were dewaxed in xylene and hydrated with gradient alcohol. After rinsing with phosphate-buffered saline (PBS), the sections were autoclaved (15 min in a microwave oven) with 0.01 M sodium citrate buffer (pH 6.0) to recover antigens. Endogenous peroxidase was inactivated with 3% hydrogen peroxide at 37 ℃ for 10 min. Description: rabbit anti-HIF-1α antibody (Bioss, Beijing, China, bs-20398R), rabbit anti-PDK1 antibody (Abcam, Cambridge, United Kingdom, ab207450), rabbit anti-TGF-β1 antibody (Bioss, Beijing, China, bs-0086R), rabbit anti-Smad2 antibody (Bioss, Beijing, China, bs-0718R), rabbit anti-Smad3 antibody (Affinity Biologicals, Beijing, China, AF6362), and rabbit Anti-alpha-SMA antibody (Bioss, bs-10196R) (1:200 dilution) were cultured overnight in a humid room at 4 °C. Antibody binding was stained with a DAB substrate kit, the cell nucleus was re-stained with hematoxylin, reverse gradient alcohol dehydration was performed, and xylene transparent and neutral resin tablets were sealed. Negative control slides were created using cattle serum albumin as a primary antibody, while all other steps and conditions remained constant.

### 2.4. Design and Synthesis of Gene Primers and RT-qPCR

#### 2.4.1. Design and Synthesis of Gene Primers

According to the HIF-1α, PDK1, TGF-β1, Smad2, Smad3, α-SMA, PCNA, Glut1, PKM2, HK-2, FN, CTFG, Collagen II, Caspase3, Caspase9, Bax, and Bcl-2 gene sequences in GenBank II, Primer Premier 6.2 was used to design HIF-1α, PDK1, TGF-β1, Smad2, Smad3, α-SMA, PCNA, Glut1, PKM2, HK-2, FN, CTFG, Collagen II, Caspase3, Caspase9, Bax, and Bcl-2 gene primers, with the ACTB gene used as an the internal reference gene. The primer sequence information is shown in Table 1, and the primers were sent to Ecorry Biological Engineering Co., Ltd. (Changsha, Hunan, China) for synthesis.

#### 2.4.2. RT-qPCR

Total RNA was extracted from the tissues and cells according to the instructions of a reverse transcription kit, and it was reversed into cDNA for RT-qPCR. The total reaction system was 20 μL/8.2 μL of sterile enzyme-free water, 1 μL of cDNA, 0.4 μL of positive and negative primers, and 10 μL of 2 × Universal SYBR Green Fast qPCR Mix. Reaction procedure: pre-denaturation at 95 °C for 30 s, denaturation at 95 °C for 5 s, and annealing at 60 °C for 35 s, for 40 cycles. The RT-qPCR reaction was performed in an Applied Biosystems 7500 fluorescence quantitative PCR (Bio-rad, Shanghai, China), and the obtained values were standardized with the internal reference ACTB. The RT-qPCR results were analyzed using the 2^−ΔΔCt^ method.

### 2.5. Preparation of Protein Samples and Western Blot

#### 2.5.1. Preparation of Protein Samples

##### Preparation of Tissue Protein Samples

Next, 0.1 g of the kidney molecular samples from the adult yak and cattle was placed in a centrifugation tube. After the tissue was completely cut using ophthalmic scissors under high pressure, 1 mL of RIPA lysate was added (10 µL PMSF was added according to the ratio of RIPA:PMSF = 100:1), and the tissue was cracked on ice for 30 min. Centrifugation was conducted at 12,000 rpm and 4 ℃ for 15 min. The supernatant was removed, the protein concentration was determined using the BCA quantitative method, and the protein concentration per sample was adjusted to 2.0 µg/µL.

##### Preparation of Cell Protein Samples

The yak and cattle RIFs kept at normal and low oxygen levels were washed 3 times with 2 mL of PBS, then 500 µL of RIPA lysate was added (adding 5 µL of PMSF according to the ratio of RIPA:PMSF = 100:1), and the next operation was the same as that described in Section 2.5.1 Preparation of Cell Protein Samples.

#### 2.5.2. Western Blot

After PAGE gel with different concentrations was prepared according to the molecular weight of the protein, the same amount of protein sample was added, the concentrated glue was run at 80 V then transferred to a PVDF membrane at 120 V, and finally sealed at room temperature with a TBST solution containing 5% skim milk for 2 h. Description: rabbit anti-HIF-1α antibody (1:500, Bioss, bs-20398R), rabbit anti-PDK1 antibody (1:1000, Abcam, ab207450), rabbit anti-TGF-β1 antibody (1:500, Bioss, bs-0086R), rabbit anti-Smad2 Antibody (1:1000, Bioss, bs-0718R), rabbit anti-Smad3 antibody (1:1000, Affinity, AF6362), rabbit Anti-alpha-SMA antibody (1:1000, Bioss, bs-10196R), rabbit anti-PCNA antibody (1:1000, Bioss, bs-2007R), rabbit anti-CollagenII Antibody (1:1000, Bioss, bs-10589R), rabbit anti-Caspase3 antibody (1:1000, Bioss, bs-0081R), rabbit anti-Caspase9 antibody (1:1000, Bioss, bs-0049R), and rabbit anti-beta-Actin antibody (1:2000, Bioss, bs-0061R) were incubated overnight at 4 °C and washed with TBST four times for 6 min each time. Then, the film was incubated at room temperature for 50 min and washed with TBST 4 times. Color-developing solution was added, and it was placed in a chemiluminescence instrument for color development. Image J was used to analyze the gray value of the obtained protein bands.

### 2.6. Isolation, Culture, and Hypoxia Treatment of Primary Renal Cells

#### 2.6.1. Isolation, Culture, and Purification of Primary Renal Cells

The recovered intact kidney was rinsed 3 times with a 37 °C saline buffer containing 5% penicillin and streptomycin. Tissue tweezers, scalpels, and ophthalmic scissors were used to remove the surface envelope and expose the medulla, followed by washing with D-Hank solution to remove as much blood as possible. The tissue was then cut (1 mm^3^) using sterilized ophthalmic scissors and washed again. The tissue fragments were sucked into a T25 cell bottle with the tip of a gun; then, the culture bottle was gently turned over, and an appropriate culture solution was added (the liquid level was lower than the top of the tissue block). After 12 h, the culture was turned over and placed in at 37 °C, 5% CO_2_ incubator at room temperature, and the liquid was changed every 40–48 h. When the yak and cattle kidney primary cells grew to 70%–80%, the tissue mass was removed, washed with PBS solution, and digested with 2 mL 0.25% trypsin. When the cell samples were observed to have larger gaps and rounded folds under microscopy, digestion was terminated by adding 2–3 mL complete medium. The wall of the culture bottle was blown repeatedly to facilitate cell suspension and inoculation in the new cell bottle. Due to the adhesion characteristics of renal interstitial fibroblasts and renal tubular epithelial cells, the renal interstitial fibroblasts were purified and obtained using the differential adhesion method. The cell suspension inoculated in the new cell bottle was placed in an incubator for 15 min and the fibroblasts first attached to the wall. After more than half of the cells in the cell suspension were visually detected and attached to the wall, the cell suspension was discarded, a new complete medium was added, and the above process was repeated 2–3 times after the cell fusion rate reached about 70%; therefore, kidney interstitial fibroblasts with a high purity could be obtained. When the cells grew to 80–90%, the cells were digested and passed through, and then they were frozen for seed preservation.

#### 2.6.2. Hypoxia Treatment of Cells

Regarding the incubators, a CO_2_ cell culture incubator (BB15, Thermo Fisher Scientific Inc., Shanghai, China) and a three-gas incubator with a 5% CO_2_ concentration (N_2_, CO_2_, O_2_, CCL-050T-8, Esco Life Sciences Group, Shanghai, China) were used. The purified cattle and yak RIFs were cultured in a hypoxic incubator at 37 °C, with 5% CO_2_ and 10% O_2_; the other conditions were the same as those of normal oxygen incubator (37 °C, with 5% CO_2_ and 20% O_2_).

### 2.7. Immunofluorescence Identification of Renal Interstitial Fibroblasts

The RIFs were inoculated into 12-well plates containing cover slides. When the cells grew to 70–80%, the medium was discarded, washed with PBS 3 times, fixed with 4% paraformaldehyde for 15 min, permeated with 1 mL 0.1% Triton X-100 at room temperature for 30 min, and washed with PBS 3 times. Then, it was sealed in 10% goat serum sealer at room temperature for 30 min. Rabbit anti-Vimentin antibody (1:200, Bioss, bs-0756R) was added and incubated at 4 ℃ overnight. Subsequently, the cells were rinsed with PBS three times and incubated at room temperature in the dark with a secondary antibody for 1 h; then, they were washed with PBS three times, and, finally, the nucleus was re-stained with DAPI. Images were obtained using a fluorescence microscope (Aus-DP 71, Tokyo, Japan).

### 2.8. Measurement of Growth Curve

The yak and cattle RIFs in a good growth state (4–7 generations) were prepared into a cell suspension, and the cells were spread in 24-well plates, with 1 mL per well, according to a cell density adjustment of 3 × 10^4^/mL. The cells were cultured in normal oxygen incubators and low-oxygen incubators (10% O_2_ concentration), and they were collected every 24 h for counting. This process was repeated in 3 holes for each sample, the average value was calculated, and a growth curve was plotted.

### 2.9. Glucose Detection

The yak and cattle RIFs in a good growth state (4–7 generations) were prepared into a cell suspension, and the cells were spread in 12-well plates, with 1 mL per well, according to a cell density adjustment of 1 × 10^5^/mL. The cells were cultured in normal oxygen incubators and low-oxygen incubators (10% O_2_ concentration), and the supernatant was collected after 6 h, 12 h, 24 h, 48 h and 72 h. A biosensor analyzer was used to determine the glucose concentration. Then, 20 μL calibration solution was added to the biosensor analyzer for calibration, and a 20 μL sample diluted 3–5-times with pure water was added to the analyzer to determine the glucose concentration. This process was repeated 3 times for each sample, and the average value was calculated.

### 2.10. Lactic Acid Detection

The supernatant sample collected from the procedure described in Section 2.9 was used for the experiment. The operation was the same as that described in Section 2.9.

### 2.11. Flow Cytometry

After being centrifugally washed with pre-cooled PBS, 1–10 × 10^5^ RIFs (including cells in the culture supernatant) of cattle and yak were collected. A 5× Binding Buffer was diluted to 1× working solution with double-steaming water, and the cells were resuspended with 500 μL 1× Binding Buffer. Then, 5 μL Annexin V-APC and 10 μL 7-AAD were added to each tube. After gentle swirl mixing, incubation was carried out at room temperature for 5 min away from light.

### 2.12. Statistical Analysis

Western Blot, RT-PCR and immunofluorescence staining were all independently repeated at least three times. For the histological analysis and immunohistochemical staining, quantification was performed using Image-Pro Plus 6.0 software. For the Western Blot analysis, quantification was performed by scanning and analyzing the strength of hybridization signals using Image J software (1.8.0_172). All examined data are expressed as mean ± standard error of measurement (SEM). The software GraphPad Prism 8.0 was used for statistical analysis, and *p* < 0.05 was considered statistically significant.

## 3. Results

### 3.1. Histological Staining Results

**Figure 1:**
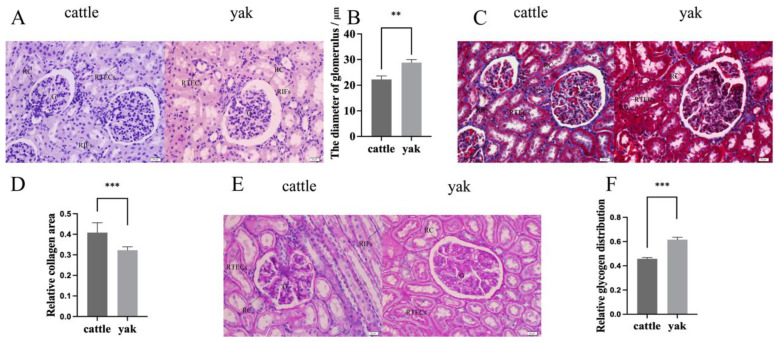
Renal histological results of adult cattle and yaks. (**A**) Kidney H&E staining results of adult cattle and yak, 400×. (**B**) Glomerular diameter of adult cattle and yaks. (**C**) Kidney Masson staining results of adult cattle and yak, 400×. (**D**) Distribution of collagen fibers in kidney tissues of adult cattle and yak. (**E**) Kidney PAS staining results of adult cattle and yak, 400×. (**F**) Distribution of glycogen in kidney tissues of adult cattle and yak. RTECs: renal tubular epithelial cells; RIFs: renal interstitial fibroblasts; G: blood vessel bulb; RC: renal sac. ***: *p* < 0.001; **: *p* < 0.01.

The H&E staining results showed that the kidneys of the adult cattle and yaks had a complete tissue structure, a good development status, and no lesions (Figure 1A). Through measurements, we found that the glomerular diameter of the adult yak kidney was significantly higher than that of the adult cattle kidney (Figure 1B). After that, Masson tri-color staining was used to analyze the distribution of collagen fibers in the kidney sections of the adult cattle and yaks. As shown in Figure 1C,D, the distribution of collagen fibers in the yak kidney tissues was significantly lower than that of the cattle kidney tissues. According to the staining results in Figure 1E, the PAS reaction in the kidney cells of the adult cattle and yak was positive, and there was a large amount of glycogen accumulation in the cytoplasm of the kidney tubular epithelial cells, glomerular cells and renal interstitial fibroblast of the cattle and yak. In addition, the proportion of glycogen distribution was measured (Figure 1F). It was found that the distribution of glycogen in the yak kidney was significantly higher than that in the cattle kidney (*p* < 0.05). These results suggest that large glycogen deposition and large glomerular diameters may be unique histological features of the yak kidney adaptation to hypoxic environments.

### 3.2. Location and Expression of HIF-1α, PDK1, TGF-β1, Smad2, Smad3, and α-SMA in Kidney Tissues of Cattle and Yak

**Figure 2:**
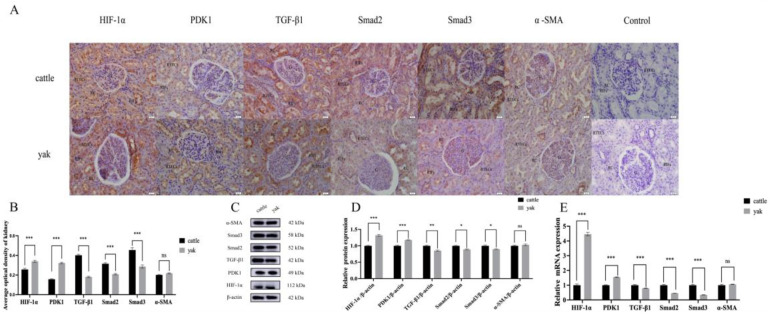
Location and expression of HIF-1α, PDK1, TGF-β1, Smad2, Smad3, and α-SMA in kidney tissues of cattle and yak. (**A**) Immunohistochemical staining results of HIF-1α, PDK1, TGF-β1, Smad2, Smad3, and α-SMA in kidney tissues of cattle and yak, 400×. (**B**) Average optical density values of HIF-1α, PDK1, TGF-β1, Smad2, Smad3, and α-SMA in kidney tissues of cattle and yaks. (**C**) Western Blot analysis of HIF-1α, PDK1, TGF-β1, Smad2, Smad3, and α-SMA in kidney tissues of cattle and yak. (**D**) Expression of HIF-1α, PDK1, TGF-β1, Smad2, Smad3, and α-SMA protein levels in kidney tissues of cattle and yak. (**E**) Expression of HIF-1α, PDK1, TGF-β1, Smad2, Smad3, and α-SMA gene levels in kidney tissues of cattle and yak. RTECs: renal tubular epithelial cells; RIFs: renal interstitial fibroblasts; G: blood vessel bulb; RC: renal sac. ***: *p* < 0.001; **: *p* < 0.01; *: *p* < 0.05; ns: *p* > 0.05.

To investigate the expression of HIF-1α, PDK1, TGF-β1, Smad2, Smad3, and α-SMA in the kidney tissues of the cattle and yaks, immunohistochemical methods were used to detect the distributions of HIF-1α, PDK1, TGF-β1, Smad2, Smad3, and α-SMA in the kidney tissues of cattle and yaks, and the results are shown in Figure 2A. Compared with the negative control, the distribution locations of HIF-1α, PDK1, TGF-β1, Smad2, Smad3, and α-SMA were basically the same, mainly distributed in the renal tubular epithelial cells and vascular bulbar endothelial cells in the kidney tissues of the cattle and yaks, with a small amount distributed in the renal interstitial fibroblasts. The results of an optical density analysis (Figure 2B) showed that the expressions of HIF-1α and PDK1 in the cattle were significantly lower than those in the yaks (*p* < 0.05), and the expressions of TGF-β1, Smad2, and Smad3 in the cattle were significantly higher than those in the yaks (*p* < 0.05). There was no significant difference in the expression of α-SMA in the kidneys of the cattle and yaks (*p* > 0.05). After that, Western Blot and RT-qPCR were used to detect HIF-1α, PDK1, TGF-β1, Smad2, Smad3, and α-SMA protein and gene expressions in the kidney tissues of the adult cattle and yaks. The results showed (Figure 2C–E) that HIF-1α, PDK1, TGF-β1, Smad2, Smad3, and α-SMA were all expressed in the kidney tissues of the adult cattle and yaks, and the expression trend was basically the same: the expressions of HIF-1α and PDK1 were higher in the yak kidney tissue, and the expressions of TGF-β1, Smad2, and Smad3 were higher in the yak kidney tissue, while the expression of α-SMA was not significantly different in the yak kidney tissue. These results demonstrate the differential expression of HIF-1α, PDK1, TGF-β1, Smad2, Smad3, and α-SMA in the kidney tissues of the cattle and yaks. This may be related to the adaptation of yaks to low-oxygen environments.

### 3.3. Isolation, Culture, and Identification of RIFs in Cattle and Yak

**Figure 3:**
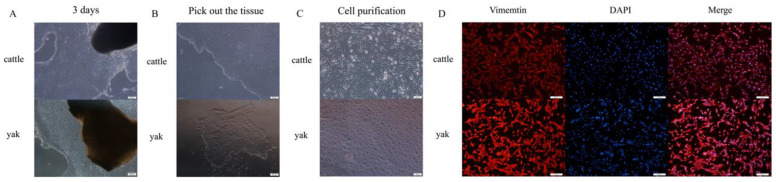
Isolation, culture and identification of RIFs in cattle and yak. (**A**) Isolation of cattle and yak kidney cells using the applanation method, 100×. (**B**) After the cells crawled out, they were removed for growth, 100×. (**C**) The isolated cultured cells were purified using the differential adhesion method, 200×. (**D**) Immunofluorescence staining results of RIFs in cattle and yak, 200×.

We used the graft method to isolate and culture renal interstitial fibroblasts. After the implant was attached to the cell vial for three days, a large number of cells crawled out (Figure 3A). Due to the complex composition of the kidney and the heterogeneity of cell lineages, the culture of primary cells produced a mixed cell population. The cell mixture mainly consisted of epithelioid RTECs and fusiform RIFs. After tissue blocks were selected and cultured, it was found that the cells had different morphological characteristics and proliferation rates. The RTECs grew in the middle, whereas the RIFs showed diffuse growth (Figure 3B). As RTECs and RIFs have different adhesion characteristics and RIFs have a faster adhesion speed, we applied the differential adhesion method for cell purification (Figure 3C). After conducting this method 2–3 times, the purified RIFs showed a spindle-shaped appearance, and they were found to be positive for vimentin after immunofluorescence identification (Figure 3D). The isolated and purified cells were used for follow-up tests.

### 3.4. Effects of Hypoxia on Glucose Metabolism in Cattle and Yak RIFs

**Figure 4:**
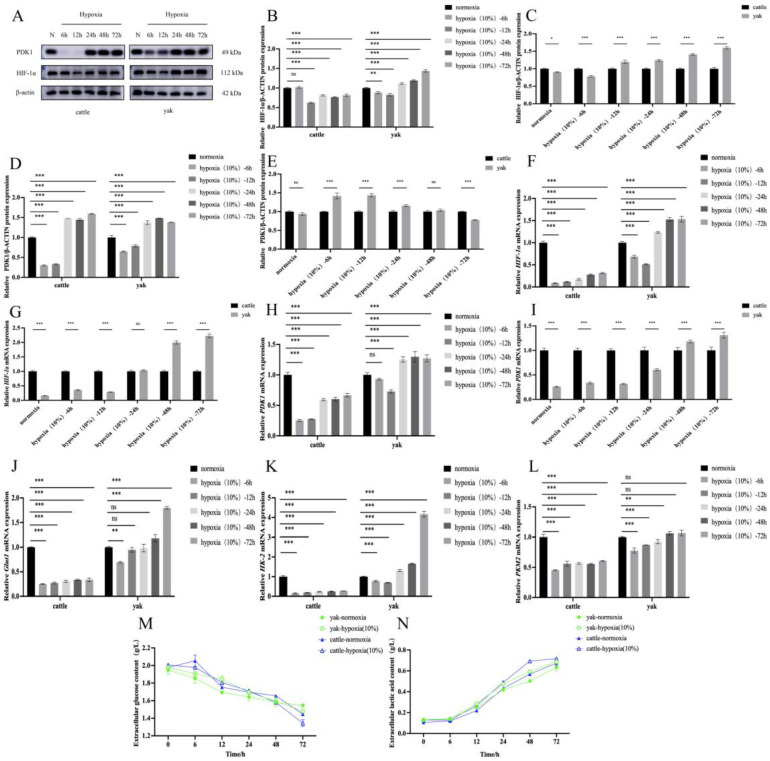
Effects of hypoxia on glucose metabolism in cattle and yak RIFs. (**A**) HIF-1α and PDK1 protein expression in cattle and yak RIFs exposed to normoxia or hypoxia for the specified times. (**B**,**D**) Relative expression of HIF-1α and PDK1 proteins at different hypoxic time in cattle and yak RIFs. (**C**,**E**) Relative expression of HIF-1α and PDK1 proteins in cattle and yak RIFs during the same hypoxic time period. (**F,H,J,K**,**L**) Relative expression of HIF-1α, PDK1, Glut1, PKM2 and HK-2 mRNAs in cattle and yaks RIFs at different hypoxic time periods. (**G**,**I**) Relative expression of HIF-1α and PDK1 mRNAs in cattle and yak RIFs during different hypoxic time periods. (**M**) Glucose consumption in the culture medium of cattle and yak RIFs exposed to normoxia or hypoxia for the specified times. (**N**) Lactate content in the culture medium of cattle and yak RIFs exposed to normoxia or hypoxia for the specified times. ***: *p* < 0.001; **: *p* < 0.01; *: *p* < 0.05; ns: *p* > 0.05.

The metabolic derangement observed in a number of chronic kidney diseases prompted us to examine whether hypoxia can affect the level of glucose metabolism in cattle and yak RIFs, so we examined the expression patterns of cattle and yak RIFs induced by hypoxia at different time periods in comparison with the expression patterns of those cultured with normal oxygen. We observed that short-term hypoxia decreased the levels of HIF-1α and PDK1 proteins (Figure 4A,B,D) and mRNA (Figure 4F,H) in the RIFs of the cattle and yak compared with the normal oxygen group. However, chronic hypoxia increased HIF-1α and PDK1 mRNA (Figure 4A,B,D) and mRNA (Figure 4F,H) levels in the RIFs of cattle and yaks. Similarly, compared with the normal oxygen group, the mRNA expression levels of Glut1, PKM2 and HK-2 genes involved in glycolysis in the RIFs cells of the cattle and yaks treated with hypoxia were consistent with the expression patterns of HIF-1α and PDK1, and long-term hypoxia could significantly promote the expression levels of these genes (Figure 4J–L). Regarding stimulation with the same hypoxia times, with short-term hypoxia, the yak RIFs could maintain the expression of PDK1 compared with the cattle RIFs, while, in long-term hypoxia, PDK1 and HIF-1α was significantly increased in the cattle RIFs (Figure 4C,E,G,I). This indicates that yak RIFs can perform normal physiological functions under short-term hypoxia stimulation, while cattle that are not adapted to a hypoxia environment can produce the energy required by cells by promoting the up-regulation of related glucose metabolism genes in order to maintain the normal state of cells after long-term hypoxia. The measurement of glucose consumption in the RIFs of the cattle and yaks (Figure 4M) and extracellular lactic acid production (Figure 4N) showed that hypoxia could significantly increase extracellular lactic acid production and glucose consumption. These glucose metabolism assessments confirmed the increase in aerobic glycolysis in the hypoxic-treated renal interstitial fibroblasts. In summary, these results suggest that hypoxia increases cellular aerobic glycolysis in renal interstitial fibroblasts.

### 3.5. Effects of Hypoxia on the Proliferation and Activation Levels of RIFs in Cattle and Yak

**Figure 5:**
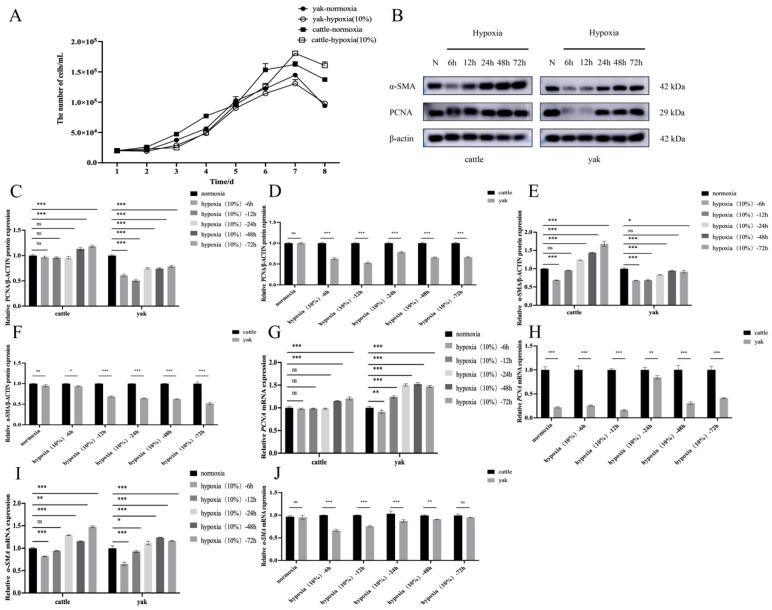
Effects of hypoxia on the proliferation and activation in cattle and yak RIFs. (**A**) RIFs proliferation in cattle and yaks was measured daily by cell counter under normal oxygen or hypoxia (10%O_2_). (**B**) PCNA and α-SMA protein expression in cattle and yak RIFs exposed to normexia or hypoxia for the specified times. (**C**,**E**) Relative expression levels of PCNA and α-SMA proteins at different hypoxic time in cattle and yak RIFs. (**D**,**F**) Relative expression levels of PCNA and α-SMA proteins in cattle and yak RIFs during the same hypoxia period. (**G**,**I**) Relative expression levels of PCNA and α-SMA mRNA at different hypoxic time in cattle and yak RIFs. (**H**,**J**) Relative expression levels of PCNA and α-SMA mRNA in cattle and yak RIFs during the same hypoxic period. ***: *p* < 0.001; **: *p* < 0.01; *: *p* < 0.05; ns: *p* > 0.05.

The activation and proliferation of renal interstitial fibroblasts are the core factors in the development of renal fibrosis after various injuries. Based on this, we adopted a model of hypoxia-treated renal interstitial fibroblasts to explore the effects of hypoxia on the proliferation and activation of renal interstitial fibroblasts. We cultured cattle and yak RIFs in an incubator with a 10% oxygen concentration to detect their degree of proliferation and activation under hypoxia. We first examined the proliferation of the renal interstitial fibroblasts in hypoxia-induced fibrotic kidneys. The growth curve results (Figure 5A) showed that there was no significant difference between the yak RIFs at 10% O_2_ and normal oxygen, while low oxygen significantly increased the proliferation of the cattle. The WB results (Figure 5B–F) showed that short-term hypoxia could significantly inhibit the proliferation and activation of the cattle and yak RIFs, while long-term hypoxia could significantly increase the proliferation and activation of the cattle RIFs; however, it was found that hypoxia could significantly increase the expression of PCNA and α-SMA proteins in the cattle RIFs compared with the yak RIFs. The RT-qPCR results (Figure 5G–J) were consistent with the WB results. Short-term hypoxia could significantly inhibit the proliferation and activation of the cattle and yak RIFs, while long-term hypoxia could significantly increase the proliferation and activation of the cattle and yak RIFs, but it was found that, compared with the yak RIFs, hypoxia could significantly increase the expression of PCNA and α-SMA in the cattle RIFs. Together, these results confirm that myofibroblast activation is accompanied by an increase in proliferation.

### 3.6. Effects of Hypoxia on Fibrosis Level of Cattle and Yak RIFs

**Figure 6:**
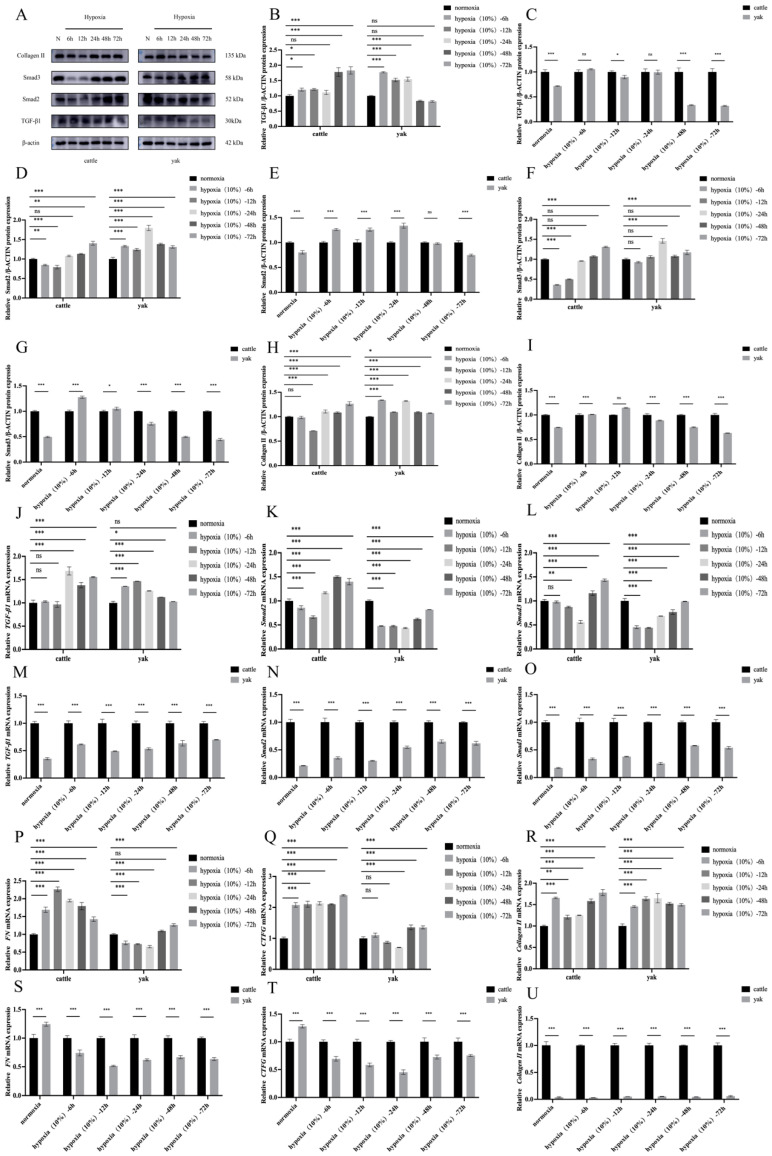
Effect of hypoxia on fibrosis in cattle and yak RIFs. (**A**) TGF-β1, Smad2, Smad3, and Collagen II protein expression in cattle and yak RIFS exposed to normal or hypoxia for the specified time. (**B**,**D**,**F**,**H**) Relative expression levels of TGF-β1, Smad2, Smad3, and Collagen II proteins at different hypoxic time in cattle and yak RIFs. (**C**,**E**,**G**,**I**) Relative expression of TGF-β1, Smad2, Smad3, and Collagen II proteins in cattle and yak RIFs during the same hypoxia period. (**J**,**K**,**L**,**P**,**Q**,**R**) Relative expression levels of TGF-β1, Smad2, Smad3, FN, CTGF, and Collagen II mRNA at different hypoxic time in cattle and yak RIFs. (**M**,**N**,**O**,**S**,**T**,**U**) Relative expression levels of TGF-β1, Smad2, Smad3, FN, CTGF, and Collagen II in cattle and yak RIFs during the same hypoxia period. ***: *p* < 0.001; **: *p* < 0.01; *: *p* < 0.05; ns: *p* > 0.05.

Hypoxia is the main microenvironmental component of fibrotic tissue, and it affects various cell types in the fibrotic region through insufficient oxygen availability. However, recent studies have shown that renal interstitial fibroblasts play an important role in hypoxia signaling for the development of renal fibrosis. In this study, relevant cytokines in the cattle and yak RIFs were detected under hypoxia. WB results showed that both long- and short-term hypoxia significantly increased the expression of TGF-β1 protein compared with normal oxygen (Figure 6A,B). Compared with normal oxygen, short-term hypoxia decreased the expression of Smad2, Smad3, and Collagen II, while long-term hypoxia significantly increased the protein expression of Smad2, Smad3 and Collagen II (Figure 6D,F,H). The results showed that hypoxia could significantly promote the expression of TGF-β1, Smad2, Smad3, FN, CTFG and Collagen II mRNA (Figure 6J,K,L,P,Q,R). However, the expression of fibrotic factors in the yak renal interstitial fibroblasts was significantly lower than that in the cattle renal interstitial fibroblasts (Figure 6C,E,G,I,M,N,O,S,T,U) during the same period of hypoxia induction. Combined with prolonged hypoxia, the metabolic disorders of renal interstitial fibroblasts in both cattle and yak further demonstrate that glycolytic reprogramming essentially enhanced the pro-fibrotic activation of renal interstitial fibroblasts, and the yak renal interstitial fibroblasts were more adaptable to a 10% oxygen concentration and less prone to renal fibrosis than the cattle renal interstitial fibroblasts.

### 3.7. Effects of Hypoxia on Apoptosis of Cattle and Yak RIFs

**Figure 7:**
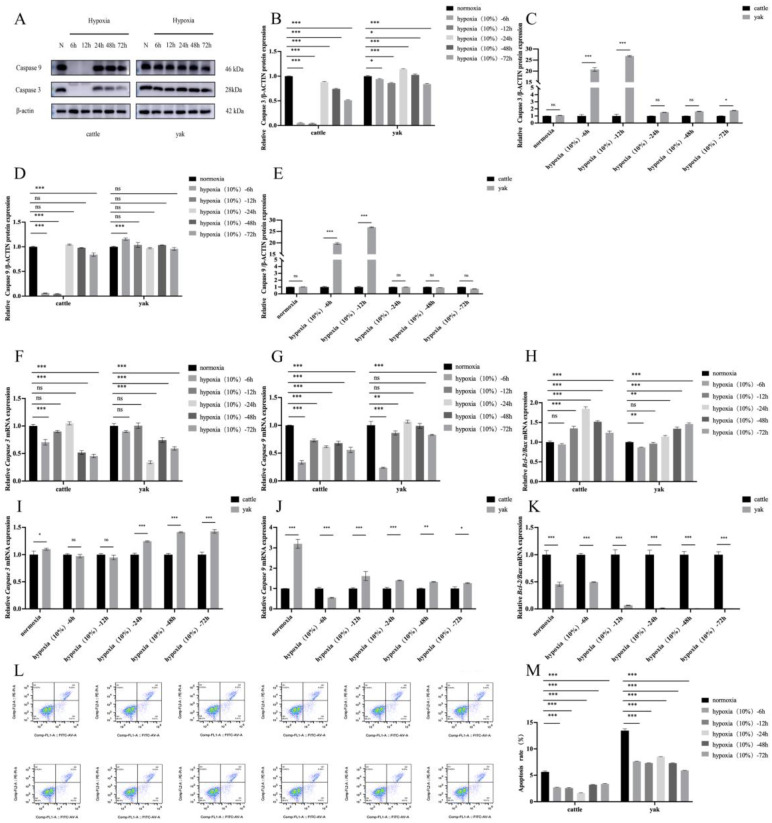
Effect of hypoxia on apoptotic in cattle and yak RIFs. (**A**) Caspase3 and Caspase9 protein expression in cattle and yak RIFs exposed to normoxia or hypoxia for the specified times. (**B**,**D**) Relative expression of Caspase3 and Caspase9 proteins at different hypoxic time in cattle and yak RIFs. (**C**,**E**) Relative expression of Caspase3 and Caspase9 proteins in cattle and yak RIFs during the same hypoxic time period. (**F**,**G**,**H**) Relative expression of Caspase3, Caspase9 and Bcl-2/Bax mRNAs at different hypoxic time in cattle and yak RIFs. (**I**,**J**,**K**) Relative expression of Caspase3, Caspase9 and Bcl-2/Bax mRNA mRNAs in cattle and yak RIFs during the same hypoxic time periods. (**L**) Distribution of apoptosis of cattle and yak RIFs exposed to normoxia or hypoxia for specified times. (**M**) Apoptosis rates of cattle and yak RIFs exposed to normoxia or hypoxia for specified times. ***: *p* < 0.001; **: *p* < 0.01; *: *p* < 0.05; ns: *p* > 0.05.

Previous studies have shown that hypoxia can significantly promote the proliferation and inhibit the apoptosis of various types of cells. Our previous results showed that hypoxia could significantly promote the proliferation of cattle kidney interstitial fibroblasts and inhibit that of yak kidney interstitial fibroblasts. Therefore, we examined the effect of hypoxia on the apoptosis of kidney interstitial fibroblasts. The WB (Figure 7A–E) results showed that with extension of hypoxia culture time, the renal interstitial fibroblasts of the cattle and yaks showed an anti-apoptotic trend, as indicated by the Caspase3 and Caspase9 protein levels being lower than those of the cells with normal oxygen. The mRNA levels (Figure 7F–K) showed the same trend, Caspase3 and Caspase9 showed a downward trend, and the anti-apoptotic index Bcl-2/Bax was higher in the hypoxic culture than in the normal oxygen culture. The results in Figure 7L–M also show that the renal interstitial fibroblasts of the cattle and yaks had a lower apoptosis rate in the hypoxic environment. In conclusion, these results confirm that yak renal interstitial fibroblasts are more suitable for long-term exposure to a 10% oxygen concentration than cattle renal interstitial fibroblasts.

## 4. Discussion

Long-term exposure of mammals to the low-oxygen environment of the plateau predisposes them to plateau sickness represented by plateau erythrocytosis, pulmonary hypertension, and right ventricular hypertrophy, which are the most common chronic plateau sicknesses in the resident plateau population. Kidney, as one of the organs sensitive to oxygen, plays an important role in hypoxia adaptation of yaks, but its specific mechanism is still unknown. Therefore, in this experiment, we investigated the differences in the tissue structure of kidney and the expression of hypoxy-related factors in kidney interstitial cells of yak and yellow cattle at different oxygen concentrations. Popescu [35] found that the glomerular diameter of newborn rats exposed to a high-oxygen environment was significantly lower than that of a control group, which is similar to our results. By comparing the diameter of the kidney tubules of cattle and yaks, we found that, in a low-oxygen environment, the diameter of the kidney tubules of the yaks was significantly higher than that of the cattle. Meanwhile, Tufro-McReddie [36] found that administering 1–3% O_2_ to rats could significantly promote the proliferation of endothelial cells and the formation of tubules and blood vessels. Glycogen is an important substance, as it allows animals to carry out their daily activities, and cells rely heavily on anaerobic glycolysis to maintain the supply of ATP in a hypoxia environment [37]. Moreover, studies have shown that the exposure of cells to a low-oxygen environment can lead to the accumulation of glycogen [38,39]. PAS staining showed that the distribution of glycogen in the kidney tissue of the yak was higher than that in the kidney tissue of the cattle. These results indicate that, in a hypoxic environment, yaks do not develop pathological changes of renal fibrosis; this may be due to the morphological changes that have occurred in yaks in low-oxygen environments due to long-term natural selection. Additionally, the higher PAS-positive reaction indicates that hypoxia may promote glycogen deposition in yaks. It is speculated that the higher amount of glycogen in the kidney provides the energy required for yaks to maintain their daily activities in the low-oxygen environment of the plateau, but the specific mechanism needs to be further investigated.

In 1924, Warburg discovered that, compared with normal cells, cancer cells have a unique ability to ferment glucose into lactic acid, even when sufficient oxygen is available [40]. This process is now thought to be a key mechanism in cancer growth, known as the “Warburg effect.” To date, it has been widely used in cancer research, and it has been found that related glycolysis inhibitors provide effective treatment for cancer [41]. However, recent studies have shown that abnormal glycolysis is not specific to cancer but is a common feature of other non-cancer diseases, including kidney disease [42]. The kidney adapts to a hypoxic environment by regulating factors that promote angiogenesis, erythropoiesis, and glycolysis. As an important hypoxic adaptation factor, HIF-1α adapts to a hypoxic environment by stimulating angiogenesis, erythropoiesis, glycolysis, iron metabolism, etc., to accelerate oxygen transport and regulate energy demand [17,43,44]. The IHC results showed that HIF-1α was mainly distributed in the cytoplasm of the renal tubular epithelial cells and vascular endothelial cells, and the expression level of HIF-1α was higher in the kidney tissues of the yak than in those of the cattle. In addition, hypoxia-treated cattle and yak RIFs demonstrated a significantly increased expression of HIF-1α protein and mRNA. This suggests that hypoxia can promote HIF-1a expression. Studies have shown that the activation of HIF-1α can also cause the up-regulation of the target gene PDK1, glucose transporter-1 (GLUT 1), glycolysis-related enzymes, and glycogen synthesis [45,46]. This is consistent with our study, which found that the expression of PDK1 was higher in the yak kidney tissue and that hypoxia could promote the expression of PDK1 and glycolysis-related enzymes (Glut1, PKM2 and HK-2). The expression of PDK1 was consistent with that of HIF-1α. PDK-1 is an essential metabolic switch induced by HIF-1α, which directs the conversion of pyruvate to lactic acid and provides a key metabolic adaptation for hypoxic cells [32,33]. Our results also show that the hypoxia-treated cattle and yak RIFs demonstrated higher levels of glucose consumption and lactic acid production. This suggests that hypoxia caused changes in glucose metabolism. This energy change has been confirmed not only in hypoxia-induced renal fibrosis, but also in unilateral ureteral obstruction models and TGF-β1-induced renal fibrosis models. It has been found that myoblast activation in the kidney is related to the enhancement of renal glucose absorption and lactate secretion, and it can be weakened by blocking glycolysis using 2DG treatment [25,26].

Although all cell types in the kidney are involved in the pathogenesis of renal fibrosis, numerous studies have shown that fibroblasts that acquire myofibroblast phenotypes and produce a large number of stromal matrix components after activation are the main stromal-producing cells [16,18,47]. Therefore, we used renal interstitial fibroblasts as a model to study the effect of hypoxia on renal fibrosis. We used two types of renal interstitial fibroblasts, one from plain cattle (cattle) and the other from plateau cattle (yak), for normal and hypoxic cultures. During fibrosis, the transformation of renal interstitial fibroblasts into muscle fibroblasts usually involves two main processes: proliferation and extracellular matrix synthesis. In our study, we found that the cattle RIFs cultured with low oxygen had higher levels of proliferative activity and increased extracellular matrix synthesis (Figure 5). Moreover, the levels of proteins and genes also showed that the RIFs were activated along with proliferation (Figure 5) and were proportional to the related fibrotic factors (Figure 6). Cell proliferation and extracellular matrix synthesis both force fibroblasts to produce the ATP necessary to maintain the normal physiological function of cells, which corresponds to our results, demonstrating that hypoxia can promote an increase in glucose metabolism in cattle and yak RIFs. This suggests that alterations in glucose metabolism are a feature of renal myofibroblast activation during renal fibrosis. We measured not only the proliferative capacity but also the apoptosis of RIFs. The results showed that long-term hypoxia could significantly inhibit the apoptosis of cattle and yak RIFs. These results suggest that renal fibrosis induced by hypoxia is accompanied by changes in the glucose metabolism of RIFs, promoting proliferation and activation, as well as anti-apoptotic levels. Thus, our findings of metabolic reprogramming present in hypoxia-treated interstitial myofibroblasts of cattle and yak kidneys can help us to better understand the metabolic alterations associated with organ fibrosis. They also underscore the critical importance of comprehensively understanding the cellular and molecular links between accelerated myofibroblast differentiation and cell-type-specific glycolytic metabolism during fibrotic disease progression.

Almost all major organs and tissues are susceptible to irreversible and fatal fibrosis. If the involvement of hypoxia-induced glycolytic reprogramming of fibroblasts is not limited to major organ fibrosis but is clinically relevant to other types of fibrosis, this further emphasizes the need for a better understanding of metabolic alterations associated with hypoxia and organ fibrosis. It has been shown that inhibition of PDK1 with small interfering RNAs or glycolysis inhibitors can shift cardiac cell metabolism from glycolysis to mitochondrial respiration [30], and it has also been found that targeting the HIF-1a/PDK 1 axis in fibroblasts can serve as a key metabolic regulatory locus in both pulmonary and cardiac fibrosis [20,34]. And in renal fibrogenesis, this previously undescribed metabolic regulatory site may prove to be a novel therapeutic target for the clinical translation of hypoxia-induced renal fibrosis or other hypoxia-related diseases.

## 5. Conclusions

This study focused on the differential regulation of hypoxia adaptation in the kidneys of cattle and yak for the first time. To explore the differences of related genes in response to changes in glucose metabolism. The results show that, compared with the kidney tissues of cattle, the kidney tissues of yak had a larger glomerular diameter and higher proportion of glycogen distribution. The relative expression levels of HIF-1α and PDK1 in the adult yak kidney tissues were significantly higher than those in the cattle (*p* < 0.05). The relative expression levels of TGF-β1, Smad2 and Smad3 in the adult yak kidney tissues were significantly lower than those in the cattle kidney tissues (*p* < 0.05). Long-term hypoxia can promote changes in the cell phenotype, including promoting the proliferation, activation, glucose consumption, lactic acid production, and anti-apoptosis of kidney interstitial fibroblasts in cattle and yaks. This study further revealed the regulatory relationship between PDK 1, HIF-1α and the TGF-β1/Smad signaling pathways in hypoxia acclimation, at high altitudes, providing basic information for the prevention and treatment of renal fibrosis induced by hypoxia, but the mechanism underlying the blocking of hypoxia-induced renal fibrosis still needs further research. Our next step will be to target the HIF-1a/PDK 1 axis to investigate how yaks are protected from renal fibrosis in a hypoxic environment, to provide a physiological for revealing the hypoxic adaptation of yak kidneys.

## Figures and Tables

**Table 1:** Primer sequence information.

Primer	Upstream Primer Sequence	Downstream Primer Sequence
HIF-1α	5′-GGCGCGAACGACAAGAAAAA-3’	5’-GTGGCAACTGATGAGCAAGC-3’
PDK1	5’-GCAAAATCACCAGGACAGCC-3’	5’-CGGATAAACACCTTTGTCAGCAT-3’
TGF-β1	5’-GCGGACTACTACGCCAAGGA-3’	5’-GCTGTGCGAGCTAGACTTCATTT-3’
Smad2	5’-TGCTGGCTCAGTCCGTTAAT-3’	5’-TTGTTACCGTCTGCCTTCGG-3’
Smad3	5’-GGGTGGATTTGGGGAAGAG-3’	5’-GGTTTGCTTTCGTGTTTTGG-3’
α-SMA	5’-CAATGGCTCTGGGCTCTGT-3’	5’-CCTCTTTTGCTTTGTGCTTCA-3’
PCNA	5’-CTTGAAGAAAGTGCTGGAGGC-3’	5’-TTGGACATGCTGGTGAGGTT-3’
Glut1	5’-TTCATCCCAGCCCTGTTGC-3’	5’-GGTTCTCCTCGTTGCGGTTA-3’
PKM2	5’-AAAGGTCCTGACTTCCTGGTG-3’	5’-GCGGATGAAAGACGCAAAC-3’
HK-2	5’-GCCTCCAAACAAAACTAGACGA-3’	5’-ACGGTATCATTCACTACAGCCAC-3’
FN	5’-GGGACCACGCAGAACTATGA-3’	5’-TCCACGACCATTTCCAACAC-3’
CTFG	5’-CTCCAAGCCTATCAAGTTTCAGC-3’	5’-AAGGGTGGTGGTTCTGTGGG-3’
Collagen II	5’-CTCAAGAAGGCTCTGCTCATCC-3’	5’-ATAGTCTTGCCCCACTTACCG-3’
Caspase3	5’-TGTCAAACAACAGCAATGACGA-3’	5’-CAGCACAAACATCACAAAACCA-3’
Caspase9	5’-GGTTGATGGTCACCGTTTTCC-3’	5’-CTGTTCATAGGCACTGTTTTCTTC-3’
Bcl-2	5’-GATGACCGAGTACCTGAACCG-3’	5’-GACAGCCAGGAGAAATCAAACA-3’
Bax	5’-CCTTTTGCTTCAGGGTTTCAT-3’	5’-CGCTCAGCTTCTTGGTGGAT-3’
β-actin	5’-ACTGTTAGCTGCGTTACACCCT	5’-TGCTGTCACCTTCACCGTTC-3’

## Data Availability

All data presented in this study are available on request from the corresponding authors.
